# Surgical Cases

**Published:** 1859-02

**Authors:** C. Brackett

**Affiliations:** Rochester, Fulton Co., Indiana


### ARTICLE IV.

### SURGICAL CASES.

BY C. BRACKETT, OF ROCHESTER, FULTON CO., INDIANA.

*Dislocation of Humerus of eleven weeks.*—Mrs. Wann, of Tippecanoe, Marshall Co., Indiana, came with her husband to me, February, 1857, with dislocated humerus. At the time of the accident, eleven weeks before, she had a surgeon (?) to see it, who undertook, or supposed he had effected, a reduction. Two or three weeks later, finding the arm still useless, and suffering much, she sent for me. I did not go. Her husband then took her to Laporte, when reduction was again undertaken by two good surgeons, assisted by (I supposed from her description) Jarvis' adjuster. This operation also failed.

Eleven weeks, lacking two days, or seventy-five days after the injury, she came to me and gave a history of the case. Found the head of the humerus down and forward; and as the case had been treated by skillful operators, I called in, to see the case and to assist, Drs. Howes and Gould, of Rochester, and Dr. South, all of Tippecanoe town. I gave chloroform. After seating her in a chair, and using a sheet fastened to a bar across a door for counter extension, made extension from the humerus with two ropes, doubled, and fastened to a strong bar across the opposite door. These ropes were in charge of Drs. Howes and Gould, and were tightened by twisting with a stick, in the manner of tightening the blade of a wood saw. As often as one rope was twisted, the other was drawn taut and twisted, when the slack was taken out of the first and twisting repeated. Thus steady extension (the steadiest that can be applied) was applied for four hours. At the end of this time I had the satisfaction of *knowing* that the head of the bone was right. I kept her arm bandaged, so that, during sleep, it could not be thrown out. At the end of a few months the arm was entirely restored to perfect usefulness.

*August 18th*, 1858.—J. Swartwout, aged about forty, sent for me. Found him vomiting; in severe pain, whole abdominal region "seemed on fire," as he expressed himself; had intense burning pain in epigastrium, more particularly. Pulse small, frequent, quick, 130 per minute; hands and feet cold, head and body hot; rejected from the stomach whatever he took; wanted quarts of water. I gave him opium grs. ii., pulv. gum acacia grs. iv., repeated in fifteen minutes; both doses rejected, though but little water taken. Then gave opii gr. i., morph. sul. gr. ss., pulv. liquorice, in very little water; and when he must vomit, as much water as he could take. He rejected two of these doses. Burning pain in stomach not relieved. Then gave opii grs. ii., morph. gr. ss., capsicum grs. iv., with water. Within ten minutes the *burning* pain ceased, vomiting quieted, and sleep restored. Gave occasionally, next day, opium, liquorice, and prepared chalk, in small doses, and directed twenty of the Sappington pills* to be taken, one at a time, at intervals of one hour; and as he was up and about the house, did not see him again. Do physicians, as a general rule, put as much confidence in large doses of opium and active stimulants, in cases of sporadic cholera, or simulated gastritis, as they ought? Do they follow Sydenham's treatment, or add fuel to fire in the commencement of the disease, by cathartics and irritant "alteratives?"

* SAP. PILLS.—℞ Qui. gr. i.; Myrrh, Pulv. Ex. Liquorice, aa, gr. ss.

